# Healthy Eating Index-2015 in relation to risk of metabolic dysfunction-associated fatty liver disease among US population: National Health and Nutrition Examination Survey 2017–2018

**DOI:** 10.3389/fnut.2022.1043901

**Published:** 2023-01-04

**Authors:** Wei Zhang, Xinyue Wang, Jiale Huang, Siyi Wang, Qing Yao, Hongwei Li

**Affiliations:** ^1^School of Public Health, Xiamen University, Xiamen, China; ^2^Xiamen Clinical Research Center for Cancer Therapy, Department of Nutrition, Zhongshan Hospital (Xiamen), Fudan University, Xiamen, China; ^3^Department of Clinical Nutrition, The First Affiliated Hospital of Nanchang University, Nanchang, China; ^4^Department of Nutrition, Zhongshan Hospital, Fudan University, Shanghai, China

**Keywords:** metabolic dysfunction-associated fatty liver disease, HEI-2015, NHANES, association, cross-sectional analysis

## Abstract

**Background:**

Metabolic dysfunction-associated fatty liver disease (MAFLD) is a serious chronic disease in the US. Dietary patterns provide good guidance for the prevention of chronic diseases. The Healthy Eating Index (HEI-2015) is a dietary pattern based on the dietary characteristics of the US.

**Objective:**

Since the relation between HEI-2015 and MAFLD is unclear, this study examined their associations using the US National Health and Nutrition Examination Surveys (NHAENS) during 2017–2018.

**Methods:**

This study included data from 4,062 participants aged ≥20 years, without viral hepatitis or pregnancy. MAFLD is defined as hepatic steatosis with one or more of the following: (1) overweight or obesity (body mass index ≥25 kg/m^2^); (2) type 2 diabetes; or (3) two or more other metabolic risk abnormalities. HEI-2015 scores were calculated from food intake information collected by the 24-h meal review method. The relationship of HEI-2015 with MAFLD was calculated using survey-weighted logistic regression analysis after adjusting for sex, age, race, education level, smoking status, alcohol use, levels of C-reactive protein, Aspartate Aminotransferase, Alanine Aminotransferase, a body shape index, minutes of sedentary activity, levels of cholesterol and glucose, energy take, drugs use, hypertension, and diabetes.

**Results:**

When compared to the study population with no MAFLD, the patients with MAFLD showed a lower weighted mean HEI (48.0 ± 0.6). HEI-2015 was inversely associated with MAFLD in the fully adjusted model [Q4 vs. Q1, OR = 0.567 (0.407–0.790), *P* = −0.002]. Among the 13 HEI-2015 components, total vegetables, greens and beans, total fruits, whole fruits, and whole grains were negatively associated with MAFLD, while added sugars were positively associated with MAFLD. This inverse association was consistent in subgroups of the participants stratified by sex, age, education level, race, body shape index, minutes of sedentary activity, hypertension, and diabetes.

**Conclusion:**

A higher HEI-2015 is associated with a lowered risk of MAFLD which is more obvious among participations who were women, young, Mexican Americans, with higher education, and with no hypertension or diabetes.

## Introduction

Non-alcoholic fatty liver disease (NAFLD) is a rapidly progressing chronic disease of the liver with an estimated prevalence of 28.0–52.34 per 1,000 persons in different countries (1, 2). The majority of patients with NAFLD also have certain features of the metabolic syndrome, such as central obesity, hypertension, dyslipidemia, and the metabolic syndrome that could increase the risk of hepatic fibrosis and lead to hepatocellular carcinoma, cirrhosis, or a higher risk of mortality (3). In 2020, a classification of metabolic dysfunction-associated fatty liver disease (MAFLD) was proposed based on the diagnosis of hepatic steatosis, while incorporating other markers of metabolic abnormalities such as insulin resistance, high-sensitivity reactive protein, and other metabolic risk factors for pathological progression (4, 5). It has been shown that compared with NAFLD, MAFLD has a higher sensitivity (91.7 vs. 73.0%), while specificity was almost identical (27.1 vs. 29.7%) (6), and was a better predictor of the risk of death in the American adult population (7).

About one-third of adults in the United States have fatty liver (8). Due to the lack of effective therapeutics and inefficient policies to evaluate the prevalence of MAFLD, the economic burden of healthcare in the United States is expected to increase. Unhealthy dietary habits and a sedentary lifestyle elevated the risk of fatty liver disease (9, 10). Excessive carbohydrate and/or fat intake has been identified as a risk factor for the development of fatty liver (11). Vitamins and polyphenols in diets lower a high level of triglycerides through multiple mechanisms, including inhibition of adipogenesis *via* downregulation of sterol regulatory element binding protein (SREBP1c) and induction of antioxidant and anti-inflammatory effects (12, 13). Consumption of nuts is negatively correlated with body weight and obesity, an effect achieved by the suppression of inflammation and oxidative stress, and the improvement of cholesterol metabolism, vascular function, as well as gut microbiome (14, 15). Nonetheless, the overall diet quality cannot be reflected by a single food or nutrient, the “diet/diet quality index” tool assesses a population's adherence to a specific pattern or set of recommendations, which reflects the population's long-term eating habits and has guiding significance for the prevention and development of chronic diseases (16). The Healthy Eating Index (HEI-2015) has been used to assess whether various food combinations meet the Dietary Guidelines for Americans (DGA2015-2020). HEI-2015 is scored from 0 to 100 with the higher score representing the higher quality of the diet (17). The score is calculated by a sum of 13 components and is used to examine the diet quality and health outcomes (prospective and cross-sectional associations among obesity, non-alcoholic fatty liver disease, and cardiovascular diseases) (17–19).

However, there are few studies on the association of HEI-2015 with NAFLD which reported that diet quality is inversely associated with NAFLD (20, 21). While the correlation between HEI-2015 and MAFLD remains unexplored, it is unclear whether the HEI-2015 could be used as a valid dietary tool to study the relationship between diet and MAFLD. Therefore, in the current study, we investigated the relationship between HEI-2015 and the risk of MAFLD by using data from the American National Health and Nutrition Examination Survey (NHANES).

## Methods

### Data source and study participates

**Figure 1:**
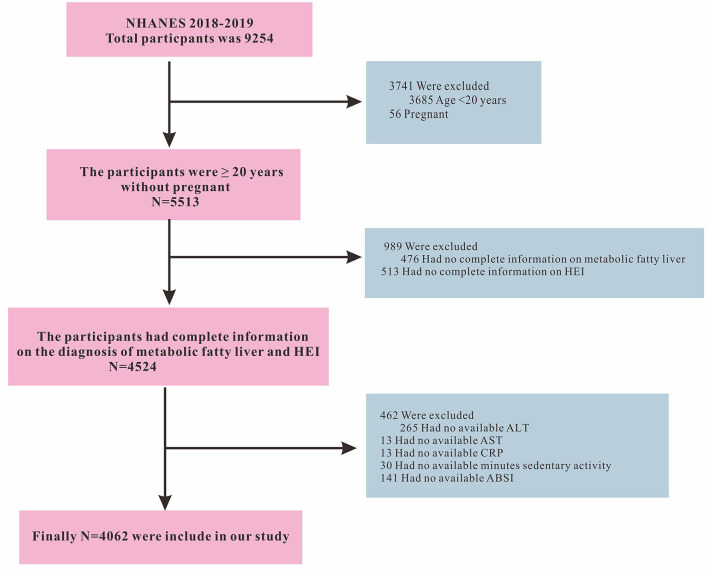
Flowchart of study participates.

The data included in this retrospective study was retrieved from the NHANES database (https://www.cdc.gov/nchs/nhanes/index.htm). The data collection was conducted by the U.S. Centers for Disease Control and Prevention using a stratified, multistage, and probability-cluster design, including oversampling of Mexican American, Non-Hispanic white, Non-Hispanic black, other Hispanic, and other races, primarily to assess their adult and child health and nutritional statuses. This study used NHANES data from 2017 to 2018. The initial search for the intended study period retrieved data on 9,254 individuals. We then excluded participants younger than 20 years (*n* = 3,685), pregnant women (*n* = 56), incomplete diagnostic indicators for MAFLD (*n* = 476), missing HEI-2015 score (*n* = 513), participants with missing Alanine Aminotransferase (ALT, *n* = 265), Aspartate Aminotransferase (AST, *n* = 13), C-reactive protein (CRP, *n* = 13), number of minutes of sedentary activity (*n* = 30), and body shape index (ABSI, *n* = 141) information. Finally, a total of 4,062 participants were included in this study. The inclusion and exclusion criteria of the study participants are shown in Figure 1. The Ethics Review Board of the National Center for Health Statistics approved the NHANES protocol and informed consent was obtained from all participants (22). This study used information from the public database which was publicly available elsewhere (18, 23).

### Diagnosis of MAFLD

NHANES 2017–2018 is the only publicly available survey database for liver fibrosis assessment by Fibroscan^®^ and has been used in the studies of MAFLD (23). Hepatic steatosis was defined by the controlled attenuation parameters (CAP), and was stratified into grades S1–3 with the thresholds of CAP for S1–S3 being 302, 331, and 337 dB/m, respectively (24). Hepatic stiffness is measured using liver stiffness measurements (LSM) and stratified into F2–4 grades with the threshold of LSM for F2–4 being 8.2, 9.7, and 13.6 kPa, respectively. Significant steatosis and stiffness were diagnosed as grades greater than S1 and F2 (24). In this study, participants with a fasting time of <3 h, with less than 10 complete LSM readings, or a liver stiffness interquartile (IQR) range/median LSM of more than 30% were deemed as failed FibroScan^®^ measurements and were excluded from further analysis (23).

Metabolic dysfunction-associated fatty liver disease is defined as hepatic steatosis with the presence of one or more of the following conditions: (1) overweight or obesity (body mass index ≥25 kg/m^2^); (2) type 2 diabetes; or (3) two or more other metabolic risk abnormalities, which were (1) blood pressure ≥130/85 mmHg or specific drug treatment; (2) overweight or obesity (body mass index ≥25 kg/m^2^); (3) plasma triglycerides ≥150 mg/dL or specific drug treatment; (4) plasma high-density lipoprotein-cholesterol < 40 mg/dL for men and < 50 mg/dL for women or specific drug treatment; (5) prediabetes (fasting glucose 100–125 mg/dL or hemoglobin A1c (HA1c) 5.7–6.4%; (6) homeostasis model assessment of insulin resistance score ≥2.5; and (7) plasma CRP level >2 mg/L (23).

### HEI-2015

The USDA-recommended HEI-2015 was used to assess adherence to the 2015–2020 DGA dietary guidelines. Dietary intake data were obtained from two 24-h recall interviews by NHANES. The first interview was conducted face-to-face at the Mobile Examination Center (MEC) and the second interview was conducted by telephone 3–10 days later. Dietary intake was estimated using the mean of two 24-h recall data. Energy and nutrient intake for each food or beverage was calculated using the Food and Nutrient Database for Dietary Studies (FNDDS), and food groups were determined by the United States Department of Agriculture (USDA) Food Pattern Equivalence Database (FPED). HEI-2015 was designed and scored from 0 to 100, which was derived from the sum of 13 components (17). First, nine adequacy components include total fruits, whole fruits, total vegetables, greens and beans, total protein foods, seafood and plant proteins (each 0–5 points), whole grains, dairy, and fatty acids (each 0–10 points). Second, four moderation components include sodium, refined grains, added sugars, and saturated fats (each 0–10 points). A higher HEI-2015 score indicates better diet quality. Supplementary Table 1 shows the details of the HEI-2015 scoring criteria.

### Other covariates

#### Demographic characteristics

Self-reported demographic variables included age (years), sex (male/female), race/ethnicity (Mexican American, other Hispanic, non-Hispanic white, non-Hispanic black, non-Hispanic Asian, or other race, including multiracial), and education level (<11th grade, High school graduate, Some college, College graduate, or above).

#### Body measurement

Trained health technicians obtained various body measurements in a mobile examination center (MEC), including height, weight, and waist circumference. ABSI is calculated from weight, height, and waist circumference, the formula is as follows:

$$\begin{array}{l} ABSI\left(m^{\frac{11}{6}}Kg^{-\frac{2}{3}}\right)=waist\left(m\right)\times weight^{-\frac{2}{3}}\left(kg\right)\times height^{5/6}\left(m\right) \end{array}$$

#### Biochemical indicators

Serum samples were processed, stored, and shipped to the University of Minnesota Advanced Research Diagnostic Laboratory (ARDL) in Minneapolis for analysis (25). The detailed laboratory methodology could be found at https://wwwn.cdc.gov/Nchs/Nhanes/2017-2018/BIOPRO_J.htm#LBDSGLSI and https://wwwn.cdc.gov/Nchs/Nhanes/2017-2018/HSCRP_J.htm#LBDHRPLC.

1. Aspartate Aminotransferase catalyzes the reaction of alpha-ketoglutarate with L-aspartate to form L-glutamate and oxaloacetate.
2. The method to measure ALT catalyzes the reaction of alpha-ketoglutarate with L-alanine to form L-glutamate and pyruvate.
3. The method to measure glucose utilizes an enzymatic method which is an endpoint reaction that is specific to glucose.
4. The method used to measure cholesterol is an enzymatic method where esterified cholesterol is converted to cholesterol by cholesterol esterase.
5. The C-reactive protein level is measured by a two-reagent, immunoturbidimetric system. The specimen is first combined with a Tris buffer, then incubated. The second reagent (latex particles coated with mouse anti-human CRP antibodies) is then added. Turbidity is measured at a primary wavelength of 546 nm (secondary wavelength of 800 nm).

### Lifestyle

The Smoking-Cigarette Use (variable name prefix SMQ) dataset provides information on the history of cigarette use. Smoking status was classified as never smoker (never smoked 100 cigarettes in the lifetime), smoked some days, or smoked every day. The alcohol used information was collected from dietary interview-Total Nutrient Intakes, First Day (DR1TOT_J). Minutes of sedentary activity from the section of the Physical Activity Questionnaire (variable name prefix PAQ) was based on the Global PAQ.

### Diseases and medications

The diagnostic criteria for hypertension are doctor diagnosis as hypertension, or SBP ≥ 140 mmHg or DBP ≥ 90 mmHg, or use of hypertension medication. The diagnostic criteria for diabetes (DM) are doctor diagnosis as diabetes, glycohemoglobin HbA1c (%) > 6.5, fasting glucose (mmol/l) ≥7.0, random blood glucose (mmol/l) ≥11.1, 2-h OGTT blood glucose (mmol/l) ≥11.1, or use of diabetes medication or insulin. The diagnostic criteria for impaired fasting blood glucose (IGF) are: 1) doctor diagnosed as IGF, 2) random blood glucose 5.6–6.9 mmol/l. The variable drug represents whether to take prescription for cholesterol.

### Statistical methods

The study used the sampling weights to account for the planned oversampling of specific groups as recommended by NHANES. All analyses were sample-weighted and accounted for the complex stratified, multistage, cluster sampling design of NHANES (26, 27). Continuous variables were expressed as the survey-weight mean ± standard error (SE). Categorical variables were expressed as counts (survey-weight percentages). Baseline characteristics between the two groups (without MAFLD and MAFLD) were compared using survey-weighted linear regression for continuous variables and survey-weighted Chi-square test for categorical variables.

The relationship of HEI-2015 with MAFLD was investigated using survey-weighted logistic regression which developed several models. Model 1 was unadjusted for any variables. Model 2 was adjusted for age and sex. Model 3 was adjusted for age, sex, race, education, smoking, alcohol, CRP, AST, ALT, ABSI, minutes of sedentary activity, total cholesterol, glucose, drug, energy, hypertension, and DM. The *P*-value for the trend was obtained by fitting a logistic regression model. The same method was used to explore the relationships between the 13 HEI-2015 components and MAFLD. To better explore the association between HEI-2015 and MAFLD, multivariable logistic regression was conducted to explore HEI-2015 as continuous and categorical variables (divided into quarters). Finally, we further used stratified logistic regression models for sensitive analyses according to potential confounding factors in Table 1.

**Table 1:** Characteristics of participants by categories of MAFLD: NHANES 2017–2018.

**Characteristics (weighted)**	**Non-MAFLD**	**MAFLD**	***P*-value**
*N*	1,718	2,344	
Energy (kcal)	2097.9 ± 29.2	2271.7 ± 21.4	0.005
HEI_2015	50.6 ± 1.0	48.0 ± 0.6	0.006
Age (years)	43.9 ± 0.7	51.4 ± 0.7	< 0.001
**Sex (%)**			
Male	765 (42.6)	1,243 (54.3)	< 0.001
Female	953 (57.4)	1,101 (45.7)	
**Race (%)**			
Mexican American	218 (5.7)	348 (10.6)	< 0.001
Other Hispanic	211 (6.8)	187 (6.5)	
Non-Hispanic white	830 (66.2)	706 (63.1)	
Non-Hispanic black	603 (12.1)	364 (9.2)	
Other races	451 (9.3)	310 (10.3)	
**Education (%)**			0.004
< 11th grade	294 (9.1)	445 (10.6)	
High school graduate	404 (26.0)	578 (29.0)	
Some college	551 (29.2)	787 (32.7)	
College graduate or above	468 (35.7)	534 (27.8)	
ABSI (m^11/6^kg^−2/3^)	0.081 ± 0.000	0.083 ± 0.000	< 0.001
ALT (μ/l)	19.4 ± 0.6	26.5 ± 0.7	< 0.001
AST (μ/l)	21.4 ± 0.6	23.0 ± 0.5	0.072
Cholesterol (mmol/l)	4.8 ± 0.0	5.0 ± 0.1	0.034
Glucose (mmol/l)	5.1 ± 0.0	5.9 ± 0.1	< 0.001
CRP	2.6 ± 0.2	4.8 ± 0.2	< 0.001
Minutes of sedentary activity	346.2 ± 8.8	356.8 ± 7.9	0.160
**Smoking (%)**			< 0.001
No	1,349 (81.3)	1,974 (84.3)	
Some days	57 (2.8)	96 (4.2)	
every day	312 (16.0)	274 (11.4)	
Alcohol (g)	11.2 ± 0.8	11.8 ± 0.7	0.640
**Hypertension (%)**			< 0.001
No	1,153 (76.2)	1,037 (47.9)	
Yes	565 (23.8)	1,307 (52.1)	
**Diabetes (%)**			< 0.001
No	1,469 (90.4)	1,435 (65.9)	
IFG	90 (4.9)	213 (10.5)	
Yes	159 (4.7)	696 (23.6)	

Additional sensitivity analyses were performed to count: (1) the HEI-2015 extreme values that were less than 1% and greater than 99% as 1 and 99%; and (2) the energy extreme values that were less than 1% and greater than 99% as 1 and 99%.

All analyses were performed using R software, version 4.0.3 (R Foundation for Statistical Computing, Vienna, Austria). Significance was set for a two-sided *P*-value at < 0.05 indicating the significance for all the analyses.

## Results

### Participants' characteristics

The baseline characteristics of participants are shown in Table 1. When compared to the study population without MAFLD, the patients with MAFLD population showed a higher weighted prevalence of men, old age, Mexican American, no smoking, hypertension, DM, lower level of education, and longer sedentary time. The weighted mean HEI (48.0 ± 0.6) was significantly lower (*P* = 0.006).

### Analysis of associated of HEI-2015 with MAFLD

The survey-weighted linear regression analysis showed that HEI-2015 was negatively correlated with MAFLD in the fully adjusted model (Model 3) (OR = 0.985; 95%CI −0.976 to 0.993). Compared with the Q1 quartile of the HEI-2015 population, the Q4 quartile had a MAFLD prevalence in Model 3 (OR = 0.567; 95%CI 0.407–0.790). *P*-values for the trend were significant in all three models (Table 2). Compared with the Q1 quartile of the HEI-2015 population, the Q4 quartile had a fibrosis prevalence in Model 3 (OR = 0.542; 95%CI 0.309–0.951; Supplementary Table 2).

**Table 2:** Association of Healthy Eating Index 2015 with MAFLD.

**Exposure**	**Model 1^a^**	**Model 2^b^**	**Model 3^c^**
HEI-2015 (continuous)	0.987 (0.979, 0.995) 0.004	0.981 (0.972, 0.990) < 0.001	0.985 (0.976, 0.993) 0.002
**Quartile of HEI-2015**			
Q1 (12.83–39.16)	1.0	1.0	1.0
Q2 (39.16–48.38)	0.857 (0.678, 1.082) 0.174	0.765 (0.590, 0.990) 0.043	0.747 (0.563, 0.991) 0.044
Q3 (48.38–58.74)	0.835 (0.613, 1.138) 0.229	0.739 (0.530, 1.030) 0.070	0.741 (0.476, 1.154) 0.170
Q4 (58.74–97.88)	0.631 (0.476, 0.838) 0.004	0.493 (0.358, 0.680) < 0.001	0.567 (0.407, 0.790) 0.002
*P*-value for trend	0.008	0.001	0.008

a No-adjusted model: adjusted for none.

b Minimally adjusted model: adjusted for sex, age.

c Fully adjusted model: adjusted for sex, age, race, education, smoking, alcohol, CRP, Aspartate Aminotransferase (AST), Alanine Aminotransferase (ALT), ABSI, minutes of sedentary activity, cholesterol, glucose, drug (take the prescription for cholesterol), energy, hypertension, diabetes.

The result of survey-weighted linear regression of HEI-2015 components demonstrated that scores of total vegetables, greens and beans, total fruits, fatty acids, and added sugars were all significantly associated with MAFLD in Model 3. The individual components of HEI-2015 that showed a negative association with MAFLD include total vegetables (OR = 0.939; 95%CI 0.866–0.994), greens and beans (OR = 929; 95%CI 0.895–0.963), total fruits (OR = 0.920; 95%CI 0.870–0.980), whole fruits (OR = 0.924; 95%CI 0.872–0.980), and whole grains (OR = 0.969; 95%CI 0.942–0.970), whereas intake of foods with added sugars showed a positive association (OR = 0.962; 95%CI 0.930 to 0.996; Table 3).

**Table 3:** Association of HEI-2015 components with MAFLD.

**HEI-2015 components**	**Model 1^a^**	**Model 2^b^**	**Model 3^c^**
**Adequacy components**			
Total vegetables	0.949 (0.890, 1.011) 0.096	0.944 (0.887, 1.004) 0.062	0.939 (0.886, 0.994) 0.034
Greens and beans	0.932 (0.887, 0.978) 0.008	0.930 (0.883, 0.980) 0.010	0.929 (0.895, 0.963) < 0.001
Total fruits	0.948 (0.896, 1.004) 0.066	0.914 (0.863, 0.969) 0.005	0.920 (0.870, 0.970) 0.003
Whole fruits	0.952 (0.903, 1.003) 0.064	0.923 (0.871, 0.978) 0.011	0.924(0.872, 0.980) 0.011
Whole grains	0.976 (0.947, 1.005) 0.100	0.955 (0.928, 0.982) 0.004	0.969 (0.942, 0.970) 0.031
Dairy	0.980 (0.949, 1.012) 0.202	0.984 (0.955, 1.015) 0.279	0.999 (0.970, 1.028) 0.913
Total protein foods	1.028 (0.952, 1.110) 0.447	1.004 (0.917, 1.100) 0.918	1.007 (0.907, 1.117) 0.893
Seafood and plant proteins	0.997 (0.955, 1.042) 0.893	0.980 (0.936, 1.026) 0.351	0.984 (0.941, 1.029) 0.448
Fatty acids	0.982 (0.951, 1.015) 0.258	0.977 (0.945, 1.011) 0.163	0.978 (0.947, 1.001) 0.168
**Moderation components**			
Sodium	0.997 (0.979, 1.016) 0.744	0.992 (0.972, 1.012) 0.407	1.015 (0.997, 1.033) 0.093
Saturated fats	0.985 (0.960, 1.011) 0.213	0.987 (0.961, 1.013) 0.285	0.987 (0.955, 1.019) 0.380
Refined grains	0.972 (0.943, 1.002) 0.068	0.962 (0.932, 0.993) 0.022	0.984 (0.949, 1.002) 0.345
Added sugars	0.974 (0.951, 0.997) 0.031	0.964 (0.940, 0.989) 0.010	0.955 (0.925, 0.985) 0.006

a No-adjusted model: adjusted for none.

b Minimally adjusted model: adjusted for sex, age.

c Fully adjusted model: adjusted for sex, age, race, education, smoking, alcohol, CRP, Aspartate Aminotransferase (AST), Alanine Aminotransferase (ALT), ABSI, minutes of sedentary activity, cholesterol, glucose, drug (take prescription for cholesterol), energy, hypertension, and diabetes.

### Subgroups analysis

The effect sizes of HEI-2015 and MAFLD in different subgroups of the study population after adjustment of other covariates were analyzed. In the population who were female, aged < 40 or 40–65 years, Mexican Americans, with a high level of education (≥high school graduate), ABSI < 0.080 or between 0.080 and 0.084, some days smoking or everyday smoking, minutes of sedentary activity 240–420 or ≥420, no hypertension, and no DM, the HEI-2015 significantly correlated with MAFLD. Race with HEI-2015 had interaction (*P* = 0.007). The details are shown in Table 4.

**Table 4:** Stratified logistic regression analysis to identify variables that modify the correlation between HEI-2015 and MAFLD.

	**Model 1^a^**	**P for interaction**	**Model 2^b^**	**P for interaction**	**Model 3^c^**	**P for interaction**
**Sex**		0.041		0.038		0.058
Male	0.994 (0.985, 1.003) 0.149		0.987 (0.977, 0.998) 0.023		0.989 (0.974, 1.005) 0.154	
Female	0.982 (0.972, 0.993) 0.003		0.975 (0.965, 0.986) < 0.001		0.981 (0.971, 0.991) 0.001	
**Age (years)**		0.894		0.895		0.819
< 40	0.980 (0.969, 0.991) 0.002		0.978 (0.967, 0.989) < 0.001		0.983 (0.968, 1.000) 0.045	
40–65	0.987 (0.979, 0.995) 0.004		0.981 (0.972, 0.990) < 0.001		0.986 (0.978, 0.995) 0.003	
≥65	0.992 (0.973, 1.010) 0.353		0.993 (0.974, 1.012) 0.427		0.991 (0.972, 1.011) 0.369	
**Race**		0.003		0.002		0.046
Mexican American	0.991 (0.972, 1.010) 0.306		0.987 (0.965, 1.009) 0.205		0.985 (0.961, 1.010) 0.201	
Other Hispanic	0.994 (0.979, 1.008) 0.352		0.985 (0.972, 0.998) 0.030		0.994 (0.969, 1.019) 0.613	
Non-Hispanic white	0.977 (0.966, 0.988) < 0.001		0.969 (0.957, 0.982) < 0.001		0.978 (0.966, 0.991) 0.002	
Non-Hispanic black	1.008 (0.998, 1.018) 0.126		1.001 (0.990, 1.011) 0.910		0.990 (0.988, 1.009) 0.794	
Other races	1.008 (0.986, 1.031) 0.439		1.000 (0.981, 1.019) 0.998		1.001 (0.975, 1.027) 0.948	
**Education**		0.006		0.015		0.171
< 11th grade	1.013 (1.000, 1.026) 0.056		1.009 (0.994, 1.024) 0.225		1.000 (0.978, 1.022) 0.963	
High school graduate	0.994 (0.977, 1.010) 0.426		0.986 (0.969, 1.004) 0.114		0.978 (0.957, 1.001) 0.057	
Some college	0.988 (0.975, 1.000) 0.051		0.980 (0.967, 0.993) 0.005		0.984 (0.968, 1.000) 0.050	
College graduate or above	0.979 (0.966, 0.992) 0.004		0.973 (0.960, 0.987) 0.001		0.980 (0.968, 0.993) 0.004	
**ABSI**		0.560		0.835		0.250
< 0.080	0.988 (0.977, 0.999) 0.039		0.984 (0.971, 0.996) 0.013		0.982 (0.966, 0.998) 0.033	
0.080–0.084	0.987 (0.979, 0.995) 0.004		0.981 (0.972, 0.990) < 0.001		0.984 (0.975, 0.992) 0.001	
≥0.084	0.994 (0.983, 1.005) 0.229		0.996 (0.986, 1.005) 0.346		0.999 (0.984, 1.014) 0.875	
**Smoking**		0.312		0.516		0.583
No	0.983 (0.974, 0.992) 0.001		0.978 (0.968, 0.988) < 0.001		0.985 (0.976, 0.994) 0.003	
Some days	1.008 (0.984, 1.032) 0.498		1.009 (0.977, 1.041) 0.562		1.004 (0.975, 1.040) 0.654	
Every day	0.997 (0.981, 1.012) 0.655		0.989 (0.972, 1.007) 0.202		0.979 (0.964, 0.994) 0.011	
**Alcohol (g)**		0.633		0.402		0.498
≤15	0.988 (0.981, 0.996) 0.005		0.982 (0.973, 0.990) < 0.001		0.985 (0.977, 0.993) 0.001	
>15	0.980 (0.959, 1.000) 0.051		0.978 (0.958, 0.998) 0.033		0.982 (0.961, 1.002) 0.078	
**Minutes of sedentary activity**		0.010		0.024		0.406
< 240	0.993 (0.984, 1.003) 0.155		0.987 (0.975, 0.999) 0.031		0.990 (0.971, 1.002) 0.078	
240–420	0.987 (0.979, 0.995) 0.004		0.981 (0.972, 0.990) < 0.001		0.985 (0.976, 0.993) 0.002	
≥420	0.984 (0.976, 0.993) 0.002		0.979 (0.970, 0.988) < 0.001		0.984 (0.975, 0.992) 0.001	
**Hypertension**		0.230		0.285		0.224
No	982 (0.973, 0.992) 0.001		0.977 (0.967, 0.987) < 0.001		0.977 (0.968, 0.986) < 0.001	
Yes	0.993 (0.978, 1.009) 0.356		0.993 (0.978, 1.009) 0.377		0.999 (0.982, 1.016) 0.899	
**Diabetes (%)**		0.366		0.266		0.909
No	0.984 (0.975, 0.993) 0.003		0.979 (0.969, 0.989) < 0.001		0.983 (0.972, 0.994) 0.004	
IFG	0.990 (0.963, 1.018) 0.456		0.991 (0.966, 1.016) 0.430		1.002 (0.975, 1.030) 0.872	
Yes	0.995 (0.974, 1.017) 0.631		0.997 (0.976, 1.018) 0.727		0.994 (0.975, 1.013) 0.519	

a No-adjusted model: adjusted for none.

b Minimally adjusted model: adjusted for sex, age.

c Fully adjusted model: adjusted for sex, age, race, education, smoking, alcohol, CRP, Aspartate Aminotransferase (AST), Alanine Aminotransferase (ALT), ABSI, minutes of sedentary activity, cholesterol, glucose, drug (take prescription for cholesterol), energy, hypertension, and diabetes.

All the models are not adjusted for the variable itself in each stratification.

### Sensitivity analyses

This study used multivariate logistic regression to perform sensitivity analyses: the HEI-2015 extreme values that were less than 1% and greater than 99% were counted as 1 and 99%. All results showed that HEI-2015 was negatively correlated with MAFLD, which was consistent with the above findings. The results of sensitivity analyses were presented in Supplementary Tables 3, 4.

## Discussion

Metabolic dysfunction-associated fatty liver disease is a newly proposed diagnosis for metabolic-related fatty liver disorders which pays more attention to the impact of metabolic abnormalities during the development of the fatty liver. Studies showed that recent changes in diagnostic criteria did not affect the prevalence of the condition in the general United States population (NAFLD was 37.1%, 95%CI: 34.0–40.4 vs. MAFLD was 39.1%, 95%CI: 36.3–42.1, and kappa coefficient was 0.83–0.94) (28, 29), while the predictor of liver mortality between NAFLD and MAFLD were different (29). There was wide variability in the cut-off values for the diagnosis of liver steatosis and fibrosis due to heterogeneity in the etiology of chronic liver disease, the prevalence of steatosis, and so on (30). A recent study showed that cut-off values of CAP ≥ 102 dB/m and LSM ≥ 8.2 kPa were suitable for the diagnosis of steatosis and fibrosis in the US population. The area under the receiver operating characteristic curves (AUROCs) of 0.87 (95% CI 0.82–0.92) with a sensitivity of 0.80 (95% CI 0.75–0.84) and a specificity of 0.83 (95% CI 0.69–0.92) at a threshold of 302 dB/m selected by maximizing Youden's index. The AUROC of 0.77 (95% CI 0.72–0.82) with a sensitivity of 0.71 (95% CI 0.64–0.77) and a specificity of 0.70 (95% CI 0.62–0.77) at the threshold of 8.2 kPa selected by maximizing Youden's index (24). Therefore, cut-off values of CAP ≥ 102 dB/m and LSM ≥ 8.2 kPa were used for the diagnosis of hepatic steatosis and fibrosis, respectively. In the present study, we explored the relationship between the HEI-2015 and the risk of MAFLD, and for the first time, reported that adherence to the HEI-2015 lowers the risk of MAFLD. The risk of MAFLD in the population with the highest HEI-2015 score (58.74–97.88) is 50% of the risk of MAFLD in the population with the lowest score (12.83–39.16).

Diet quality is an important factor in reducing the risk of the development of metabolic-related diseases. The HEI Index is a dietary quality assessment system designed based on the dietary characteristics of the US population, and high scores were characterized by a high intake of plant foods (such as whole grains) and moderate intake of alcohol, and a low intake of red and processed meat, sodium, sweetened beverages, and trans fatty acids (17). The higher the HEI scores, the better the quality of the metabolic diet. The better HEIs tend to be associated with better health behaviors in individuals who complied with the diet quality index. Higher HEI scores were observed in participants who were older, married, had higher education levels, had higher levels of physical activity, and were smoking less (19, 31). In this study, a high HEI-2015 dietary index was found to be a protective factor for MAFLD after adjusting for these covariates. This is the first study to explore the relationship of HEI-2015 with MAFLD, and the result was consistent with previous studies investigating the relationship between HEI and metabolic-related diseases such as obesity, cardiovascular disease, diabetes, and non-alcoholic fatty liver disease (18, 32–35). Additionally, in the subgroup analysis, HEI-2015 also was a good indicator of the associated population characteristics, such as being young, higher educated, and more active. While our study did not show a negative relationship between HEI and MAFLD in the population with metabolic diseases (hypertension and DM), contrastingly, HEI was strongly associated with lower odds of NAFLD in those without DM in the US population but showed no association with advanced fibrosis (20). This suggests that if the disease progresses to a certain stage with other metabolic disorders, it might be necessary to combine diet, exercise, and drugs to slow the development of MAFLD. However, the number of participants with DM (*n* = 886) or hypertension (*n* = 1,940) was small in this study, which might be the reason for not observing such a negative association in the study population. In this study, we found the prevalence of MAFLD patients with DM was higher than non-MAFLD, and a systematic review and meta-analysis showed that 1 in 20 patients with T1D and 1 in 5 patients with T2D has elevated liver stiffness, which indicates diabetes as a risk factor for advanced disease (36). This warrants future research to explore diet, exercise, and drug combination effects on MAFLD among participants with DM.

The HEI components associated with MAFLD were total vegetables, greens and beans, total fruits, grains, and added sugars in this study. Among these results, a high-quality intake of total vegetables, greens and beans, total fruits, and grains was widely recognized to be associated with a reduced risk of chronic diseases. People with a high intake of these types of foods as seen in the Mediterranean diet, which is a healthy eating pattern, have a lower risk of cardiovascular and other chronic diseases (37, 38). Since the pathogenesis of MAFLD is closely related to the risk factors of cardiovascular disease, high-quality scores of these foods are protective factors that reduce the risk of MAFLD (39). Fruits, vegetables, grains, and greens and beans are rich in polyphenols, vitamins, and unsaturated fatty acids, which contain antioxidant, anti-inflammatory, and cholesterol-lowering effects (40–42), which can regulate the metabolic abnormalities in MAFLD, such as higher blood lipids, blood glucose, and CRP. The HEI-2015 scores of these components were lower in the US population, so it is necessary to strengthen the intake of these foods. Foods containing lower added sugar were associated with higher HEI scores, but for the US population, added sugars account for 89.7% of energy intake (43) whose consumption is associated with increased risk of all-cause mortality and chronic diseases such as cancer, cardiovascular disease, diabetes, hypertension, and dyslipidemia in recent prospective studies (44–46). The excessive intake of high-calorie sweetened foods and beverages is associated with overweight and obesity that can increase the risk of cancer through obesity-related mechanisms, including insulin resistance hyperinsulinemia, increased bioavailability of steroid hormones, oxidative stress, and inflammation (46, 47). According to 2013 nationally representative data, 68% of available packaged foods and beverages purchased by US households contained these caloric sweeteners (43). Therefore, controlled sugar intake is also a protective factor for the prevention of MAFLD in the American population where the prevalence of MAFLD is about 36.3–42.1% (28).

The present study results reinforce the notion that the quality of the food we eat has a profound impact on health outcomes and supports the current DGA recommendations on the importance of healthy eating patterns as a whole rather than focusing on an individual nutrient or food. However, as this is a cross-sectional study with a small sample size of the US population, stronger evidence is needed to show the guiding significance of HEI in the prevention of chronic diseases.

There are several strengths in our study. First, HEI-2015, a comprehensive dietary index, better reflects Americans' compliance with the 2015–2020 DGA. Second, the NHANES database contains a large and nationally representative sample of the adult population of the United States. Third, the study used the weight in the analysis method, to adjust for covariates in different models, and also considered the association of HEI and MAFLD in different populations, thus performing sensitivity analysis. However, our study has a few limitations. First, the cross-sectional design of our study cannot accurately reflect the causal relationship between HEI-2015 and the risk of MAFLD. Second, 24-h dietary interviews do not necessarily reflect long-term diet consumption habits. Third, although the study adjusted for many covariates, the effect of unmeasured confounders still cannot be ruled out.

## Conclusion

HEI-2015 is inversely associated with the prevalence of MAFLD, suggesting that better diet quality is associated with a lower risk of MAFLD. More interestingly, this negative relationship is more significant in the individuals who were female, young, Mexican Americans, with high-level education, had no hypertension, no DM, and those with frequent activity. Our study findings provide new insights into the diet/nutrient-based prevention strategies of MAFLD and show that improvements aiming at elevating diet quality may help to reduce the risk of developing MAFLD. Nevertheless, more high-quality prospective studies are needed to clarify the causal relationship between them.

## Data availability statement

Publicly available datasets were analyzed in this study. This data can be found at: https://www.cdc.gov/nchs/nhanes/index.htm.

## Ethics statement

The Ethics Review Board of the National Center for Health Statistics approved the NHANES protocol and informed consent was obtained from all participants.

## Author contributions

XW, HL, and QY designed the study. WZ, SW, and JH organized and analyzed the data. XW and HL contributed materials and analysis tools. WZ and JH wrote the paper. All authors contributed to the study's conception and design and have read and approved the final manuscript.
