# Supplementary material for: Healthy Eating Index-2015 in relation to risk of metabolic dysfunction-associated fatty liver disease among US population: National Health and Nutrition Examination Survey 2017–2018
Source: Front Nutr. 2023 Jan 4;9:1043901. doi: 10.3389/fnut.2022.1043901 (PMC9875296; doi:10.3389/fnut.2022.1043901)
Supplement: Supplementary file 1 [file Data_Sheet_1.docx]

**Table S1** HEI–2015 Components and Scoring Standards ^a^

| HEI-2015 Components | Minimum Scoring and Standard | Maximum Scoring and Standard |
| --- | --- | --- |
| Total Vegetables | 0 (0 cup equiv. /1000 kcal) | 5 (1.1 cup equiv. /1000 kcal) |
| Greens and Beans | 0 (0 cup equiv. /1000 kcal) | 5 (0.2 cup equiv. /1000 kcal) |
| Total Fruits | 0 (0 cup equiv. /1000 kcal) | 5 (0.8 cup equiv. /1000 kcal) |
| Whole Fruits | 0 (0 cup equiv. /1000 kcal) | 5 (0.4 cup equiv./1000 kcal) |
| Whole Grains | 0 (0 oz equiv. /1000 kcal) | 10 (1.5 oz equiv. /1000 kcal) |
| Dairy | 0 (0cup equiv./1000 kcal) | 10 (1.3 cup equiv./1000 kcal) |
| Total Protein Foods | 0 (0 oz equiv./1000 kcal) | 5 (2.5 oz equiv./1000 kcal) |
| Seafood and Plant Proteins | 0 (0 oz equiv./1000 kcal) | 10 (0.8 oz equiv./1000 kcal) |
| Fatty Acids ^b^ | 0 [(PUFAs + MUFAs)/SFAs ≤1.2] | 10 [(PUFAs + MUFAs)/SFAs ≥2.5] |
| **Moderation Components** (higher score indicates lower consumption) | | |
| Sodium | 0 (2.0 grams /1000 kcal) | 10 (1.1 grams/1000 kcal) |
| Saturated Fats | 0 (16% of energy) | 10 (8% of energy) |
| Refined Grains | 0 (4.3 oz equiv./1000 kcal) | 10 (1.8 oz equiv. /1000 kcal) |
| Added Sugars | 0 (26% of energy) | 10 (6.5% of energy) |

^a^ Intakes between the minimum and maximum standards are scored proportionately.

^b^ Ratios of polyunsaturated and monounsaturated fatty acids (PUFAs and MUFAs) to saturated fatty acids (SFAs).

**Table S2** Association of Healthy Eating Index 2015 with liver fibrosis

| Exposure | Model 1^a^ | Model 2^b^ | Model 3^c^ |
| --- | --- | --- | --- |
| HEI-2015 (continuous) | 0.985(0.974,0.997) 0.017 | 0.982(0.971,0.994) 0.007 | 0.987 (0.971,1.002) 0.081 |
| Quartile of HEI-2015 |  |  |  |
| Q1(12.83-39.22) | 1.0 | 1.0 | 1.0 |
| Q2(39.22-48.65) | 0.762(0.501,1.159) 0.184 | 0.718(0.459,1.123) 0.130 | 0.712 (0.475,1.067) 0.094 |
| Q3(48.65-58.98) | 1.029(0.702,1.509) 0.873 | 0.978(0.644,1.485) 0.907 | 1,074 (0.650,1.774) 0.170 |
| Q4(58.98-97.88) | 0.542(0.341,0.863) 0.014 | 0.473(0.290,0.772) 0.007 | 0.542 (0.309,0.951) 0.035 |
| P value for trend | 0.041 | 0.018 | 0.150 |

^a^ No-adjusted model: adjusted for None
^b^ Minimally adjusted model: adjusted for sex, age

^c^ Fully adjusted model: adjusted for sex, age, race, education, smoking, alcohol, CRP, Aspartate Aminotransferase (AST), Alanine Aminotransferase (ALT), ABSI, minutes sedentary activity, cholesterol, glucose, drug (take prescription for cholesterol), energy, hypertension, diabetes

**Table S3** Association of Healthy Eating Index 2015 with MAFLD (after deal with extreme HEI-2015 scores)

| Exposure | Model 1^a^ | Model 2^b^ | Model 3^c^ |
| --- | --- | --- | --- |
| HEI-2015 (continuous) | 0.986(0.978,0.995) 0.003 | 0.981(0.972,0.989) <0.001 | 0.984 (0.976,0.993) 0.001 |
| Quartile of HEI-2015 |  |  |  |
| Q1(12.83-39.16) | 1.0 | 1.0 | 1.0 |
| Q2(39.16-48.38) | 0.857(0.678,1.082) 0.174 | 0.765(0.590,0.990) 0.043 | 0.746 (0.561,0.992) 0.044 |
| Q3(48.38-58.74) | 0.835(0.613,1.138) 0.229 | 0.739(0.530,1.030) 0.070 | 0.740 (0.477,1.149) 0.166 |
| Q4(58.75-97.88) | 0.631(0.476,0.838) 0.004 | 0.493(0.358,0.680) <0.001 | 0.563 (0.402,0.789) 0.002 |
| P value for trend | 0.008 | 0.001 | 0.008 |

a No-adjusted model: adjusted for None

b Minimally adjusted model: adjusted for gender, age

c Fully adjusted model: adjusted for gender, age, race, education, smoking, alcohol, CRP, Aspartate Aminotransferase (AST), Alanine Aminotransferase (ALT), ABSI, minutes sedentary activity, cholesterol, glucose, drug (take prescription for cholesterol), energy, hypertension, diabetes

**Table S4** Association of Healthy Eating Index 2015 with MAFLD (after deal with extreme energy)

| Exposure | Model 1^a^ | Model 2^b^ | Model 3^c^ |
| --- | --- | --- | --- |
| HEI-2015 (continuous) | 0.986(0.978,0.995) 0.003 | 0.981(0.972,0.989) <0.001 | 0.984 (0.976, 0.993) 0.001 |
| Quartile of HEI-2015 |  |  |  |
| Q1(12.83-39.50) | 1.0 | 1.0 | 1.0 |
| Q2(39.50-48.72) | 0.857(0.678,1.082) 0.174 | 0.765(0.590,0.990) 0.043 | 0.748 (0.563,0.993) 0.045 |
| Q3(48.72-59.15) | 0.835(0.613,1.138) 0.229 | 0.739(0.530,1.030) 0.070 | 0.743 (0.480,1.152) 0.170 |
| Q4(59.15-92.10) | 0.631(0.476,0.838) 0.004 | 0.493(0.358,0.680) <0.001 | 0.565 (0.404,0.791) 0.003 |
| P value for trend | 0.008 | 0.001 | 0.008 |

a No-adjusted model: adjusted for None

b Minimally adjusted model: adjusted for gender, age

c Fully adjusted model: adjusted for gender, age, race, education, smoking, alcohol, CRP, Aspartate Aminotransferase (AST), Alanine Aminotransferase (ALT), ABSI, minutes sedentary activity, cholesterol, glucose, drug (take prescription for cholesterol), energy, hypertension, diabetes
